# Penetration and Preliminary Efficacy of a Novel Nitric Oxide-Releasing Gel for Onychomycosis

**DOI:** 10.3390/jof11110780

**Published:** 2025-10-30

**Authors:** Aditya K. Gupta, Elizabeth A. Cooper, Harmanpreet Kaur, James Martins, Simon J. L. Teskey, Chris C. Miller

**Affiliations:** 1 Mediprobe Research Inc., London, ON N5X 2P1, Canada; lcooper@mediproberesearch.com; 2 Division of Dermatology, Department of Medicine, Temerty Faculty of Medicine, University of Toronto, Toronto, ON M5S 1A8, Canada; 3 SaNOtize Research & Development Corp, Vancouver, BC V6P 3T3, Canada; hkaur@sanotize.com (H.K.); jmartins@sanotize.com (J.M.); steskey@sanotize.com (S.J.L.T.); chris@sanotize.com (C.C.M.)

**Keywords:** onychomycosis, dermatophyte, nitric oxide, antifungal

## Abstract

Onychomycosis is a therapeutically challenging fungal infection. Systemic antifungals are limited by adverse effects and drug interactions, while topical therapies may fail to achieve therapeutic nail bed concentrations. Nitric oxide (NO), a small, diffusible free radical with broad-spectrum antimicrobial activity, offers a novel approach to overcoming these barriers. We assessed the penetration and subsequent efficacy of a nitric oxide–releasing gel (NORG) in the treatment of onychomycosis. Ex vivo human nail models assessed NORG’s transungual penetration and antifungal activity via colorimetric, immunohistochemical, and microbiological assays. NORG eradicated *Trichophyton mentagrophytes* completely (0 CFU/g), outperforming terbinafine (3.58 ± 0.2 log_10_ CFU/g). In an ex vivo infection model, NORG achieved fungal clearance within 14 days, continuing to Day 30 treatment end, with no regrowth during 21 days of incubation post-treatment. Clinical data from patients with onychomycosis who received topical NORG therapy show that NORG penetrated the nail plate and nail bed, as evidenced by s-nitrosothiol accumulation and progressive discoloration. The NORG formulation demonstrates in vitro efficacy; controlled trials are warranted to fully assess clinical efficacy and safety of this NORG formulation in humans, and establish optimal treatment protocols.

## 1. Introduction

Onychomycosis is a chronic fungal infection of the nail apparatus that poses significant therapeutic challenges [1]. Current treatment modalities are limited by suboptimal efficacy and safety concerns. Systemic antifungal agents, which provide the best efficacy for moderate to severe cases, are associated with potential hepatotoxicity, drug–drug interactions, and other adverse effects [2]. Topical therapies, although safer, often fail to achieve therapeutic drug concentrations at the site of infection due to poor transungual penetration [3,4]. Topical antifungal therapy offers a favorable safety profile, with minimal systemic exposure and reduced risk of adverse events [5,6,7]. However, the efficacy of topical agents has historically been limited, with complete cure rates under 20%. Achieving sufficient drug penetration through the dense nail plate is a primary hurdle to providing therapeutic concentrations at the site of infection [8,9,10]. Furthermore, the extensive use of a narrow selection of antifungal drug classes across both clinical and agricultural domains has contributed to the emergence and spread of antifungal-resistant fungal pathogens, raising serious public health concerns [11,12,13,14,15,16,17].

Given the limitations of existing therapies and the rising burden of disease, there is a pressing need for novel antifungal agents with improved safety profiles, enhanced efficacy, and reduced potential for resistance development. Nitric oxide (NO) is an endogenous gaseous free radical that functions as a primary signaling molecule with diverse physiological roles, including regulation of vascular tone, neurotransmission, immune modulation, and wound healing [18,19,20,21,22]. NO has a small molecular weight (30 g/mol) and size (~115 pm), much less than the average antifungal drug molecule. NO also has both lipophilic and hydrophilic properties which allows it to diffuse rapidly across biological membranes.

NO exerts concentration-dependent immunomodulatory effects. At lower concentrations, NO regulates immune cell function and promotes anti-inflammatory pathways [23,24]. In contrast, at higher levels, NO and its reactive derivatives exhibit potent antimicrobial activity via nitrosylation of nucleic acids, thiol-containing enzymes, and membrane lipids, ultimately compromising microbial viability [25,26,27].

Mechanistically, NO disrupts fungal cell membranes, inhibits keratinase activity essential for nutrient acquisition within the nail bed, and promotes the formation of reactive nitrogen species and S-nitrosothiols with fungitoxic properties [28,29]. Activated macrophages synthesize NO via inducible nitric oxide synthase (iNOS) as a critical effector mechanism against pathogenic fungi [30]. However, fungal pathogens associated with onychomycosis can suppress local cellular immune responses, contributing to chronic infection and therapeutic resistance [31,32]; The exogenous application of NO, therefore, represents a promising strategy to overcome such immune evasion. The simultaneous attack on key independent targets may also reduce the potential for the development of antifungal resistance mechanisms to NO [33].

The present study investigates the potential of a novel nitric oxide–releasing gel (NORG) for onychomycosis. The chemical properties and activities of NO assist in providing antimicrobial, immunoregulatory, and biofilm dispersal properties which have been useful for the treatment of infections such as athlete’s foot and diabetic foot wounds [34,35,36,37,38]. Within the nail plate setting, NO may confer additional benefits beyond direct toxic action upon fungal structure and function. NO reaction with the abundant cysteine and disulphide bonds produces nitrosothiols, converting a favorable host-tissue niche into a hostile microenvironment [39]. In vitro testing indicates that nitrosothiols are predominantly responsible for sustained antifungal nail effects, rather than NO itself, and nitrosothiols were present through the full thickness of the nail after single application to the dorsal nail surface [39]. The existing research suggests NO/nitrosothiols have good basic nail penetration in vitro, but chemical generation of NO may vary widely with formulation. Existing published data may not be an accurate assessment of action provided by the commercial NORG formulation used in our research. Our research with this NORG formulation confirms a similar good toenail penetration and some evidence of clinical safety for human use in onychomycosis, supporting broader use of this commercially available formulation for human onychomycosis trials.

## 2. Objectives

Ex vivo testing and observational clinical data was collected to investigate the antifungal efficacy and nail-penetrating capacity of NORG, per the following objectives:

1. Does NORG penetrate nails sufficiently?
2. Does NORG provide antifungal activity after penetration?
3. Is NORG safe and effective for human use?

## 3. Materials and Methods

### 3.1. Human Nail Samples

Human great toenails were obtained from cadavers through Science Care (Phoenix, AZ, USA). All specimens were of normal thickness with no visible evidence of disease and similar light pigmentation. All specimens were stored at −80 °C until use to preserve tissue integrity.

### 3.2. Antifungal Agents

The NORG formulation was provided by SaNOtize Research and Development Corporation (Vancouver, BC, Canada) [40]. Acidified nitrite molecules begin the release of NO immediately following admixture with a hydrogel material, and release continues in a controlled manner most strongly over several hours and then diminishing as the hydrogel hydration diminishes. Occlusion of the nail prolongs the gel hydration and subsequent NO release. Once NO is released, it quickly diffuses and reacts rapidly with surrounding materials within seconds, including nail plate cysteine and disulphide bonds leading to nitrosothiol formation [39].

Terbinafine was purchased from a commercial supplier and used as a reference antifungal agent for comparative analysis. A terbinafine test formulation was made as follows: terbinafine was first dissolved at 100X final concentration in DMSO containing 5% Tween 80, after which sequential twofold dilutions were made in DMSO followed by sequential fivefold dilutions in RPMI medium to 0.002 μg/mL [41,42,43,44].

### 3.3. Fungal Strains

The dermatophyte strains used for testing were *Trichophyton mentagrophytes* (ATCC 18748, ATCC 37035), obtained from the American Type Culture Collection (Manassas, VA, USA). Stock cultures were maintained in Sabouraud dextrose broth supplemented with 25% glycerol at −80 °C. Working cultures were propagated on Sabouraud dextrose agar (SDA) and incubated at 25 °C for 7 days. To ensure ongoing viability, cultures were subcultured every 28 days and stored at 4 °C in sealed containers.

### 3.4. Testing Performed

#### 3.4.1. Colorimetric and Immunohistochemical Permeation Assays

To evaluate the ability of NORG to permeate the nail unit, an ex vivo assay using human cadaver toenails was conducted. Nails were mounted on wooden dowels and sealed along the underside using moldable clay to restrict exposure to topical application only. Each nail received a 0.25 mL dose of NORG applied to the dorsal surface once daily under the following conditions: (i) untreated control, (ii) 15-day treatment without occlusion, (iii) 15-day treatment with 8-h post-application occlusion using a silicone cot, and (iv) 30-day treatment with 8-h post-application occlusion.

Constitutive pigmentation levels were measured over the course of treatment using a skin colorimeter (MDD/CL440, C + K Khazaka, Köln, Germany) which provides an objective measure of pigment intensity through calculation of the Individual Typology Angle (ITA°) as follows:

ITA = [arctan ((L* − 50)/b*)] × 180/π

where L* indicates brightness (0 = black, 100 = white) and b* represents the blue-yellow color spectrum. Four random measurements per nail were taken at each assessment timepoint and averaged to determine mean ITA° ± standard deviation (SD). Calculated angle ranges, from greater than +55° to less than −50°, represent a spectrum from lightest to darkest color tones, respectively.

Following treatment, samples were processed for immunohistochemistry as per Finnen et al. [39]. In brief, nails were sagittally sectioned, fixed onto microscope slides, and subjected to immunohistochemical staining using 5-nitrosocysteinylcysteine-specific antibodies. Stained slides were evaluated by a board-certified pathologist, and photomicrographs were captured for documentation. Unstained sections were examined visually for macroscopic changes in color or texture.

#### 3.4.2. Ex Vivo Comparative Nail Penetration and Efficacy Assay

To assess transungual antifungal efficacy, a comparative penetration study between NORG and terbinafine was performed. Cadaver nails were hydrated in deionized water at 60 °C for 15 min, followed by sequential vortex rinsing in 70% ethanol and sterile water. Nails were then soaked in sterile water for 2 h and air-dried under sterile conditions.

The ventral nail surface was inoculated with 100 µL (1 × 10^7^ conidia/mL) of *T. mentagrophytes*, placed dorsal side down on SDA plates, and incubated at 25 °C for 8 h. Test formulations (NORG 0.125 mL; Terbinafine 0.125 mL, equivalent to 0.002 μg/mL) were applied to the dorsal surface, then nails were placed ventral side down on fresh SDA plates and incubated at 25 °C for 8 h. Post-treatment, the nails were washed three times with sterile saline, mechanically pulverized, and suspended in saline. Pulverization consisted of cutting the nail into small pieces and grinding with a mortar and pestle, with all implements pre-sterilized via autoclave. The aqueous gel should easily be removed by the saline washing, and saline would also raise the pH, neutralizing ability of the gel to generate NO. Serial tenfold dilutions were plated on potato dextrose agar (PDA) and incubated at 25 °C for 7 days to quantify viable fungal burden. Colony-forming units (CFU) per gram of nail were determined and expressed as log_10_ CFU/g ± SD. Statistical comparisons between treatment groups were conducted using a two-tailed Student’s *t*-test, with *p* < 0.05 considered statistically significant.

#### 3.4.3. Ex Vivo Onychomycosis Infection Model

To simulate chronic fungal infection of the nail unit, cadaver toenails were inoculated on the ventral surface with 100 µL (1 × 10^7^ conidia/mL) of *T. mentagrophytes*, followed by incubation on SDA plates at 25 °C for 14 days to establish infection. A subset of infected nail samples was used to confirm fungal colonization by cutting small sections, soaking in sterile saline, and plating 100 µL of supernatant onto SDA for incubation at 25 °C over 6 days.

Three (3) remaining infected nail samples were treated topically with NORG once daily for 30 consecutive days, and one (1) nail was kept as untreated control. At days 7, 14, 21, and 30, sections were excised for fungal culture as described above. Following completion of the treatment course, the nails were washed with phosphate-buffered saline (PBS) and incubated under standard conditions for 14 days to evaluate potential fungal regrowth. Nails were then pulverized, plated, and incubated for an additional 7 days to assess residual fungal viability.

#### 3.4.4. Clinical Safety and Efficacy of NORG

Prior to initiation of a clinical trial, some small-scale pilot use of NORG was performed to observe preliminary effects of daily dosing with in vivo onychomycotic toenails. A 72-year-old male with a 2-year history of great toenail onychomycosis consented to a pilot use of the NORG formulation, providing photographs for review of the treatment course and follow-up. This case is discussed, and provides some limited data in addition to two self-selected individuals with varying presentations of nail fungal infections who used the NORG formula for onychomycosis, as reported in a prior publication [45].

## 4. Results

### 4.1. NORG Fully Permeates the Nail Unit

**Figure 1:**
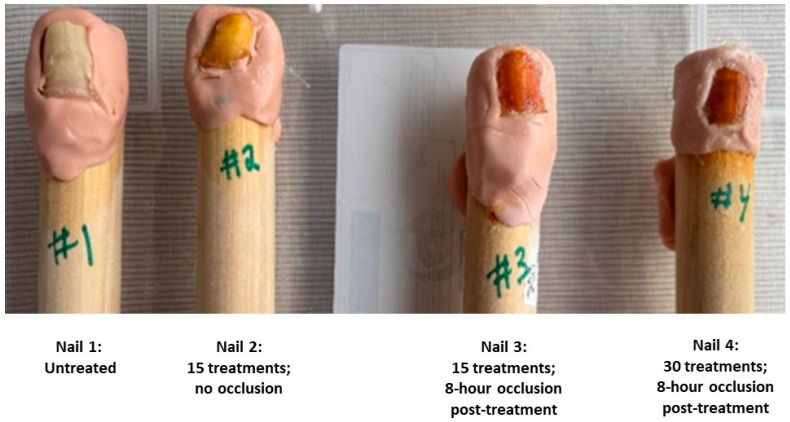
Ex vivo nails showing chromatic response to NORG exposure.

Nitric oxide (NO) released from NORG is known to induce a time-dependent chromatic change on the nail plate and surrounding epidermis, transitioning from yellow to dark red or brown with prolonged exposure. Colorimetric analysis demonstrated a relationship between the number of NORG applications and the Individual Typology Angle (ITA°), with longer exposure correlating with progressively darker nail discoloration (Table 1, Figure 1).

**Figure 2:**
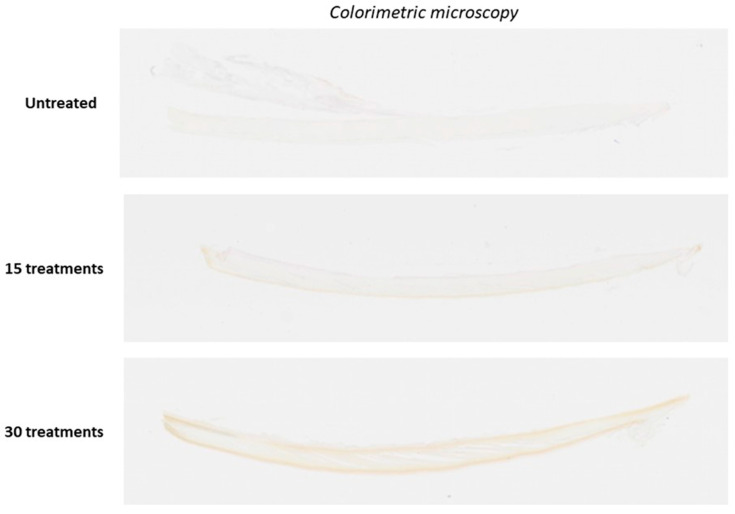
Sagittal section imaging of IHC chromatic changes.

**Figure 3:**
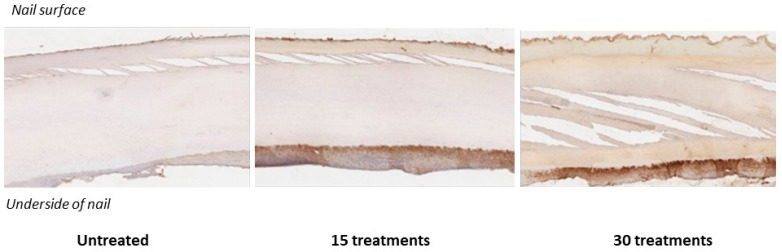
Sagittal section imaging following IHC staining.

Consistent with prior findings, immunohistochemical (IHC) staining using S-nitrosocysteine-specific antibodies confirmed the formation of S-nitrosothiols after NO permeation of the nail unit. (Figure 2 and Figure 3) Visual inspection revealed a positive correlation between the intensity of this discoloration and the degree of antibody staining, suggesting that nail color change may serve as a surrogate marker for s-nitrosothiol accumulation and, by extension, NO permeation.

An independent pathologist assessment reported positive IHC staining within the nail plate in Nails #3 and #4, and positive cytoplasmic staining of the nail bed epithelium in Nails #2, #3, and #4. Interestingly, Nail #2 which was treated for 15 days without occlusion showed nail bed staining despite the absence of detectable staining in the overlying nail plate. In contrast, Nails #3 and #4, which were occluded for 8h post-treatment with a silicone cot, demonstrated staining in both the nail bed and nail plate.

Taken together, these results provide strong evidence that NO generated from NORG effectively penetrates the healthy nail plate and diffuses into the nail bed. (Figure 3) These findings are consistent with prior work by Finnen et al. [39]. Further study is needed to confirm penetration through hyperkeratotic nails consistent with onychomycosis infection. However, Finnen et al. noted that nitrosocysteine formation occurred through the full thickness of the nail after a single application [39], while our data demonstrates increased accumulation through to the lowest nail depths with ongoing NORG treatment courses. These findings are promising as to the ability of NO/nitrosocysteine to penetrate thicker nail depths.

### 4.2. NORG Retains Antifungal Activity After Penetration

**Figure 4:**
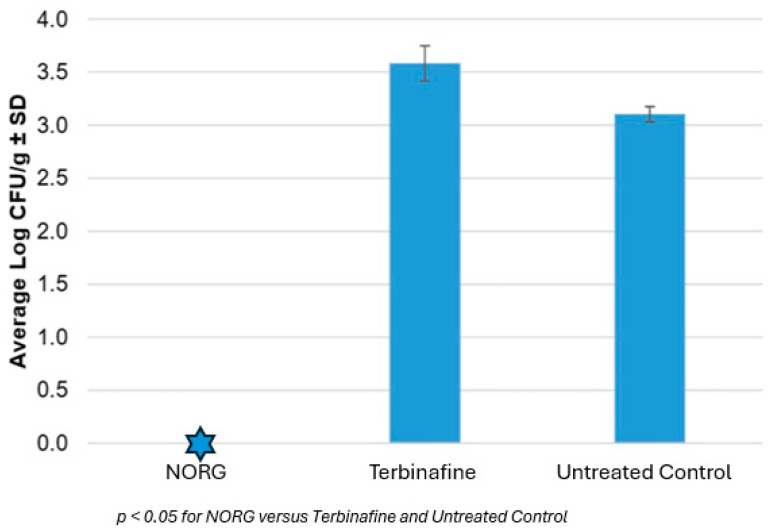
Average log CFUs/g ± S.D. for nails inoculated with *T. mentagrophytes* 1 × 10^7^ cells/mL and treated with test compounds.

In an ex vivo penetration assay, NORG demonstrated superior antifungal efficacy compared to terbinafine. (Figure 4) Untreated, inoculated nails exhibited an average fungal load of 3.10 ± 0.1 log_10_ CFU/g. NORG-treated nails showed complete fungal eradication with no detectable CFU (0 ± 0 log_10_ CFU/g), whereas terbinafine-treated nails maintained a high fungal burden (3.58 ± 0.2 log_10_ CFU/g). Statistical analysis confirmed that NORG significantly reduced fungal load relative to both the untreated control and the terbinafine group (*p* < 0.05).

These results suggest that NORG can effectively permeate the nail plate and produce antifungal activity at the site of infection, whereas terbinafine failed to demonstrate comparable penetration or activity under the same conditions.

### 4.3. NORG Inactivates Fungal Infection in an Ex Vivo Nail Model

**Figure 5:**
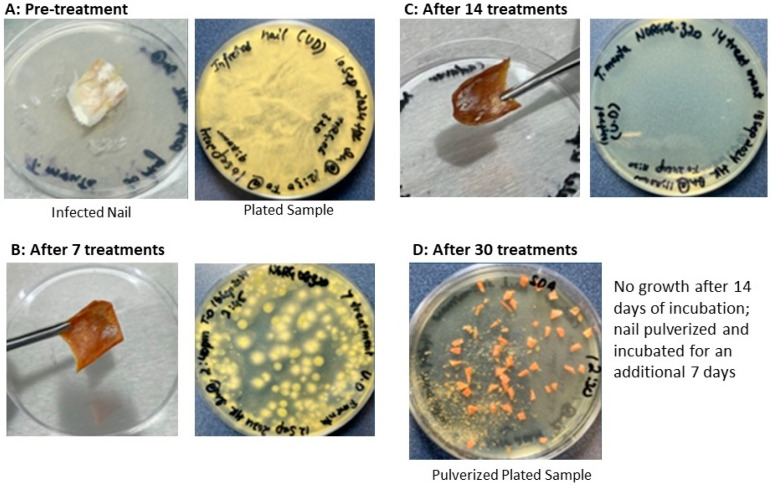
Pre- and post-treatment cultures of *T. mentagrophytes* infected nail.

An ex vivo model of onychomycosis was established by inoculating cadaveric toenails with *T. mentagrophytes*, resulting in successful colonization, confirmed visually and through culture which yielded confluent fungal growth. (Figure 5A) Treatment with NORG resulted in a marked reduction in fungal burden by day 7, and complete fungal clearance was achieved by day 14 in all tested samples. (Figure 5C) No fungal regrowth was observed after 21 days of post-treatment incubation following a 30-day course of daily NORG application. (Figure 5D) In contrast, the control developed a full lawn of growth when tested at Day 30.

These findings confirm that NORG achieves inactivation of fungal infection in the nail, suggesting that NO permeates not only the nail plate but also may effectively sterilize the nail bed. The data support the potential of NORG as a topical therapy for onychomycosis.

### 4.4. Preliminary Observations of NORG Use in Individuals with Onychomycosis

**Figure 6:**
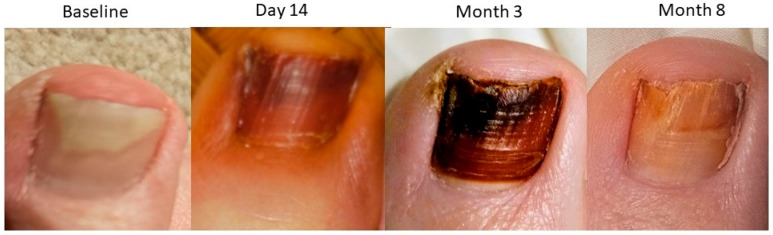
Progressive nitrosative discoloration and outgrowth across a 2-month NORG treatment with 6-month post-treatment follow-up in a 72-year-old male with 2-year history of onychomycosis.

A 72-year-old male with a >2-year history of onychomycosis affecting the right great toenail elected to undergo NORG therapy after prior treatment failures with topical agents including efinaconazole (Jublia^®^), Emtrix^®^, and Funginail^®^. A baseline dermatophyte test strip was positive for fungal infection. NORG was applied nightly, under occlusion for 6 to 8 h, for 2 months. Mycological testing was negative for fungal growth at Day 42 follow-up. The subject continued with some intermittent once-weekly applications post-Month 2 as prophylaxis. Progressively darkening discoloration, attributed to nitrosative reaction with non-viable keratinocytes, is noted in the nail across the visits and remains mildly present post-treatment as the nail grows out. (Figure 6) Some mild discoloration is also noted in the skin surrounding the nail during treatment, but does not remain post-treatment. The discoloration can be observed to progress distally as the nail grows, with no residual treatment irritation noted at any time. The patient reported no adverse events during treatment.

Similar treatment discoloration was noted in 2 patients discussed in a previous publication, indicating progressive development of nitrosocyteine in the nail plates over the treatment periods [45]. No visible treatment irritation was noted in these patients at the observation points, despite the discoloration. One patient experienced mild local stinging that resolved without recurrence upon temporary treatment interruption.

The follow-up periods for these individuals are shorter than the 12-month period recommended for observation of full great toenail outgrowth; the follow-up periods are also not sufficient in these individuals to see full removal of the treatment discoloration. The treatment discoloration complicates the evaluation of efficacy, and any future clinical trials must ensure discoloration has grown out before final efficacy determinations can be made reliably.

Progressive darkening nail discoloration indicated that NORG penetration to the nail plate/bed was occurring in these patients, as with our in vitro test results. Preliminary signs of efficacy were observed in these individuals’ cases, and in conjunction with the observations of preliminary safe use, these findings support the development of a clinical trial of safety and efficacy in a wider variety of onychomycosis cases.

## 5. Discussion

Effective topical antifungals must adequately penetrate the nail plate and enter the nail bed. Ex vivo colorimetric and immunohistochemical analyses demonstrated that NO released from NORG permeates the full thickness of the healthy human nail unit. A time-dependent reduction in ITA° values indicative of chromatic shifts to darker pigment shades was noted with increased number of treatments, and accumulation of S-nitrosothiols within the nail plate and bed confirmed deep tissue diffusion. The underlying mechanism of the observed color change, whether due to cumulative s-nitrosothiol deposition or oxidative processes, remains to be elucidated. Notably, nail bed staining in the absence of nail plate staining in the non-occluded sample suggests that NO diffusion through the nail structure may precede the pharmacological activity which leads to overt discoloration. These findings are clinically relevant, suggesting that therapeutic action may not be externally visible in early treatment phases. Furthermore, the expected change in nail colour from repeated gel applications may be useful as a marker of compliance, with respect to both drug applications and use of occlusion, in patients using NORG treatment. It remains to be seen if penetration will be as complete in hyperkeratotic nails typical of onychomycosis.

Based on the ex vivo models, NORG can provide a good antifungal effect on *T. mentagrophytes* infection following penetration of the nail structures. The antifungal effect continued during short-term post-treatment observations. These findings are consistent with NO’s known mechanism of action, including nitrosative and oxidative stress mediated via reactive nitrogen species such as peroxynitrite and S-nitrosothiols which disrupt fungal cellular respiration, membrane integrity, and DNA replication [29,39,46]. Preliminary in vitro data (unpublished) obtained during formulation testing indicated by time-kill curves that *T. mentagrophytes* was less susceptible to this NORG than *T rubrum*, hence research with the limited cadaver nails available focused on *T. mentagrophytes*.

The authors acknowledge that *T. rubrum* is the most frequently reported organism in onychomycosis cases in North America. NO has been effective in vitro against *T. rubrum*, and it is reasonable to expect that the tested formulation could adequately treat this species as well upon sufficient nail penetration [39,47]. In vitro and in vivo studies have demonstrated broad-spectrum antimicrobial activity of NO, encompassing bacteria, viruses, fungi, and yeast species [28,47,48,49,50,51,52,53,54,55,56,57]. Antifungal efficacy has specifically been observed against organisms such as *Aspergillus fumigatus*, *A. niger*, *Monilinia fructicola*, *Penicillium italicum*, and *Colletotrichum coccodes*, with notable inhibition of hyphal growth and conidial germination [58,59].

Testing in a wider population is needed to further verify safety and efficacy of applications for onychomycosis. Preliminary use of NORG in 3 individuals was well-tolerated in the daily regimens used. Its pharmacokinetic profile is characterized by a short half-life and the absence of systemic accumulation, contributing to a favorable safety margin for localized therapeutic use [60,61]. NORG penetration through the nail was demonstrated by the progressive color changes visible across the treatment period, but penetration through more-severely hypertrophic nails must be tested and may require increased dosing versus thinner nails. Evaluation of efficacy is also somewhat hampered by the treatment discoloration produced Subjects need to be followed until discoloration grows out fully to verify a visual clearance of infection. However, proximal nail fold improvements and nail outgrowth progression may be visible quickly post-treatment as the discoloration regions are removed with nail plate outgrowth. More rigorous testing of clinical and mycological efficacy is needed, using longer follow-up periods that are more aligned to nail outgrowth requirements.

Systemic absorption of topically administered NO was not evaluated here, but previous research suggests that NO absorption is minimal due to its rapid inactivation upon contact with hemoglobin, forming methemoglobin, which is subsequently reduced to nitrate and excreted renally [62]. Clinical testing of another NO gel formulation for tinea pedis, used once daily for 14 days, showed no evidence of methemoglobinemia development in any patients (Intention-to-treat population = 222 patients) [63].

These findings also suggest that NORG could address some of the fundamental therapeutic limitations in onychomycosis. By delivering active antifungal concentrations directly to the site of infection without systemic exposure, NORG bypasses issues related to hepatic metabolism, pharmacokinetic variability, and adherence challenges associated with oral therapy. Additionally, an ability to maintain antifungal effects in the nail bed post-treatment may help reduce the risk of recurrence, which is reported to occur in up to 50% of treated individuals [64].

Nonetheless, the limitations of this study must be acknowledged. The ex vivo models, while controlled and replicable, do not fully recapitulate the complexity of in vivo infected nail physiology, host immune interactions, or real-world variables such as patient compliance and comorbid conditions. Similarly, the preliminary observations of human use, although promising, included a small number of patients without control arms or standardized outcome metrics. Larger, randomized controlled trials of onychomycotic nails are needed to rigorously evaluate efficacy, optimize dosing regimens, assess long-term recurrence rates, and establish comparative performance against existing standard-of-care agents.

## 6. Conclusions

This integrated analysis of ex vivo experimentation and observational clinical cases demonstrates that nitric oxide–releasing gel (NORG) can permeate the human nail unit, and exert potent fungicidal activity against *T. mentagrophytes*. Preliminary case studies suggest a good safety profile for NORG, with some evidence of efficacy over short-term follow-up that promotes further clinical testing for onychomycosis. Given its novel mechanism of action, rapid antifungal effects, and promising safety and efficacy signals, NORG could represent a significant addition to the management of onychomycosis, particularly for patients contraindicated for systemic treatment or unresponsive to existing topical agents who need effective antifungal treatment.

In summary, nitric oxide (NO) represents a novel antifungal modality. Its exceptionally small molecular size and high diffusivity enable effective transungual penetration, while its multifaceted antimicrobial mechanisms may confer broad-spectrum activity against fungal pathogens. These properties support its potential as both a standalone and adjunctive therapy in the management of recalcitrant fungal nail infections.

## Figures and Tables

**Table 1:** Results of colorimetric analysis.

Day.	Nail #1	Nail #2	Nail #3	Nail #4
	ITA° (SD)	ITA° (SD)	ITA° (SD)	ITA° (SD)
**15**	44.0° (0.5)	15.7° (2.2)	0.75° (0.5)	−16.0° (1.8)
**30**	42.2° (2.6)	11.2° (3.0)	1.5° (1.0)	−36.5° (3.3)

ITA = Individual Typology Angle in degrees; SD = standard deviation (degrees).

## Data Availability

Data is contained within the article.
